# Thyroid and Aging or the Aging Thyroid? An Evidence-Based Analysis of the Literature

**DOI:** 10.1155/2013/481287

**Published:** 2013-09-09

**Authors:** Naveen Aggarwal, Salman Razvi

**Affiliations:** ^1^Department of Endocrinology, Gateshead Health NHS Foundation NHS Trust, UK; ^2^Institute of Genetic Medicine, Newcastle University, UK

## Abstract

Thyroid hormone production, metabolism, and action change with aging. The reference ranges for serum thyrotropin and thyroid hormones are derived mainly from younger populations. Thus, the prevalence of subclinical thyroid dysfunction is increased greatly in the elderly. However, it is unclear whether mild thyroid dysfunction in the elderly is associated with adverse outcomes. In this review, we discuss current evidence-based literature on thyroid function in the elderly and whether subclinical thyroid dysfunction in the elderly should be treated.

## 1. Introduction

As a result of declining fertility and increasing longevity, the populations of a growing number of countries are aging rapidly. Between 2005 and 2050, half of the increase in the world population will be accounted for by a rise in the population aged 60 years or over, whereas the number of children (persons under age 15) will decline slightly. Furthermore, in the more developed regions, the population aged 60 or over is expected to nearly double (from 245 million in 2005 to 406 million in 2050), whereas that of persons under age 60 will probably decline (from 971 million in 2005 to 839 million in 2050) [1].

As one ages, changes occur in all body systems including the endocrine system. These changes may be due to the amount of hormones secreted or the sensitivity of target organs. In some cases, the changes in amount of hormones secreted may be secondary to changes in target organs (e.g., LH and FSH). In addition, there may also be some change in the rate of metabolism of other hormones (e.g., increased peripheral degradation of thyroid hormones) [2].

There has been increasing interest in thyroid function in the elderly because of association of thyroid status with disability, cognitive function, cardiovascular disease risk, and longevity. The effects of overt thyroid dysfunction are well documented in all age groups. The effects of subclinical thyroid disease in elderly population are still unclear, mainly due to lack of randomized control trials (RCTs). In this paper, we evaluate the evidence about association of subclinical thyroid disease in the elderly with adverse outcomes and the evidence regarding intervention in this particular age group.

## 2. What Is “Normal Thyroid Function” in the Elderly?

There has been long standing controversy about the thyroid function test results in the elderly [3]. Serum TSH, free T4, and free T3 concentrations change with aging [4–13]. The first Whickham survey, published in 1977, showed that TSH levels did not vary with age in males but increased markedly in females after the age of 45 years. The rise of TSH with age in females was virtually abolished when persons with thyroid antibodies were excluded from the sample [14]. However, in this landmark study, the number of individuals aged 75 or more was quite small, thus limiting the ability to detect a significant increase in TSH in this age group. The 20-year follow-up Whickham survey showed that with increasing age, the incidence of positive antithyroid antibodies and hypothyroidism also increased [15]. This follow-up study, though, was unable to assess longitudinal change in serum TSH and thyroid hormones as more sensitive assays had been utilized, thus making any meaningful comparisons difficult. The larger and more recent NHANESIII survey showed that serum TSH concentrations as well as serum thyroid peroxidase (TPOAb) and thyroglobulin (TgAb) antibodies rise with age in both men and women [16]. In this study, the median TSH increased and T4 decreased after age 20 in all ethnic groups, even after excluding thyroid antibody status and other risk factors. In a subsequent further analysis, Surks and Hollowell examined the NHANESIII data which showed a progressive increase in mean, median, and 97.5 centile for TSH concentration with age in the disease-free and reference populations. This analysis suggested that the 97.5 centile is about 3.6 mIU/litre in people who are 20–39 yr of age and 5.9 and 7.5 mIU/litre in those who are 70–79 and 80 yr old and older, respectively [17]. They also demonstrated that about 70% of older patients who would be classified as subclinical hypothyroidism with TSH greater than 4.5 mIU/litre were within their age-specific reference range. Consequently, the authors have suggested that age-based reference ranges for TSH should be considered [18].

Moreover, a recent longitudinal study from Western Australia (Busselton survey), for the first time, showed that serum TSH increases (mean increase of 0.32 mU/L over 13 years) with no significant change in free T4 concentrations with aging [19]. Similarly, another longitudinal thyroid function evaluation in a very elderly subgroup (mean age 85 years) of the Cardiovascular Health Study (All Stars Study) found that serum TSH increased by 13% over an average of 13 years of follow-up associated with a 1.7% increase in FT4 and a 13% reduction in total T3 levels [20].

All the above studies (Whickham, NHANESIII, Busselton, and CVHS All Stars Surveys) have been conducted in iodine sufficient areas. Contrary to these findings, a cross-sectional study performed in an area of borderline sufficient iodine intake showed that serum TSH concentrations decreased gradually with age throughout life, whereas FT4 levels increased only in participants older than 60 years [21]. The authors hypothesized that this finding could be a result of development of thyroid autonomy after longstanding iodine insufficiency although iodine status itself was not measured in this cohort. An earlier study performed in a previously iodine deficient area in Germany showed a lower reference range (0.25 to 2.12 mIU/litre) for serum TSH in people without thyroid disease [22].

Moreover, there are studies which show that subclinical hypothyroidism and subclinical hyperthyroidism may correct spontaneously over time. A study by Parle et al. showed that over 1 year follow-up, TSH returned to normal spontaneously in 5% of people aged 60 years or more with subclinical hypothyroidism and in 76% of patients with low but detectable TSH [23]. In another similar and larger study, albeit in adults across all age groups, over a 5-year period, TSH normalized without any intervention in more than 50% of patients with elevated or decreased serum TSH level [24]. 

Setting an upper limit for the normal value of TSH for a population has implications for the diagnosis of subclinical hypothyroidism as well as related issues such as screening, association with comorbidities, especially cardiovascular risks, and treatment. Thus, the current data support the view that serum TSH rises slightly with aging, but the data on free T4 is conflicting. Prospective large studies are required to confirm whether age-specific reference ranges should be utilized when reporting thyroid parameters.

## 3. Thyroid Status and Cardiovascular Risks

A number of studies have shown an association between hypothyroidism and atherosclerosis; one of the early ones being the study by Vanhaelst et al. in 1967, which showed a greater prevalence and severity of coronary atherosclerosis in the hypothyroid patients as compared to controls [25]. 

Subclinical hypothyroidism (SCH) is associated with impaired left ventricular diastolic function at rest, systolic dysfunction on effort, and enhanced risk for atherosclerosis and myocardial infarction [26]. SCH has been shown to be associated with adverse lipid profile [27–29], increased carotid intimal thickness [28], and endothelial dysfunction [29, 30], with all these parameters reversing with levothyroxine replacement.

A fundamental issue to consider is the effect of age on the association between SCH and ischemic heart disease (IHD). The Cardiovascular Health Study cohort showed that in people above age of 65, SCH is not associated with increased risk of cardiovascular disease, mortality, or heart failure, although the latter was significantly higher in those with serum TSH > 10 mIU/litre [31, 32]. A similar result was obtained in an analysis from the Health, Aging, and Body Composition Study of individuals aged 70−79 years [33]. In a meta-analysis performed by one of the authors of this review, subjects were divided into those below and those above the age of 65 years [34]. Interestingly, the incidence and prevalence of IHD and cardiovascular mortality was higher in the younger age group but not in the group above 65 years of age. Another recent study has shown no association between persistent or transient subclinical hypothyroidism and incident IHD, heart failure, or cardiovascular death [35]. In this study, the authors used 2 models to define persistent SCH: in one model, there were two readings of TSH in SCH range in samples taken 2 years apart, and in the second model, there were 4 readings in similar range over a period of up to 8 years. In both of these models, the findings were similar: not showing any association between SCH and cardiovascular disease. Furthermore, an observational study of *real life *practice performed from data obtained from the United Kingdom General Practitioners Research Database (GPRD) showed that treatment of SCH with levothyroxine was associated with fewer IHD events in younger individuals (40–70 years), but this was not evident in older people (>70 years) [36]. The Leiden 85+ study in which 599 people were followed from age of 85 years through age 89 years (mean follow up 3.7 years) showed that increasing levels of TSH and decreasing levels of free thyroxine, both representing lower thyroid function, were associated with a survival benefit mainly due to reduced IHD events [37].

There have been a number of observational studies reporting an association of subclinical hyperthyroidism with IHD [38, 39], atrial fibrillation [31, 39–41], and cardiac dysfunction [32, 42]. A recent large study by Collet et al. [43] pooled individual data from 10 prospective cohort studies and concluded that endogenous subclinical hyperthyroidism is associated with increased risks of total and IHD mortality, and incident AF, with highest risks of IHD mortality and AF when TSH level is lower than 0.10 mIU/litre. 

These results indicate that a higher TSH in the elderly may not have any adverse effects on the cardiovascular system and may even be protective. On the other hand, a low TSH is associated with adverse vascular outcomes. Large adequately designed trials are required to confirm that the association between serum TSH levels and adverse vascular outcomes in the elderly is causal. At this juncture, one European study has commenced recruitment investigating whether levothyroxine treatment improves outcomes in SCH patients aged 65 years or older [44].

## 4. Thyroid Status and Cognitive Function

Overt hypothyroidism is associated with cognitive impairment and depression [45–48]. There are few studies that have explored the relationship between thyroid function in euthyroid elderly people and cognition [37, 49–53]. All studies have shown a relationship between thyroid function and cognition, but the results are conflicting regarding the most sensitive marker (TSH, T4, or T3). Furthermore, there is variation regarding the particular domain of cognition affected by changes in thyroid hormone concentrations.

A number of studies have shown the adverse effect of SCH on cognition in younger age groups [54–56]. However, in the elderly population, the studies give conflicting results. One study in individuals with mean age of 74 years showed that people with SCH had worse performance on verbal recall and MMSE, but working memory and processing speed were unaffected [57]. The PAQUID survey of individuals aged 65 yr or more showed that increased TSH levels were significantly linked with the presence of symptoms of depression but not with impairment of cognitive function [58]. There have been other studies which also do not support any association between SCH and cognitive impairment [59–62]. 

There have been few RCTs investigating improvement in cognition with levothyroxine replacement in SCH. Three small RCTs in middle aged individuals showed improvement in cognitive function with levothyroxine replacement therapy in people with SCH [63–65]. Two larger RCTs with longer followup have not shown any benefit in cognition with levothyroxine replacement [66, 67]; the latter study was specifically in the elderly population aged 65 years or over.

A recent systematic review has considered the association of subclinical hyperthyroidism with dementia [68]. It concluded that there is a substantial body of evidence to support the association between subclinical hyperthyroidism and cognitive impairment. It also concluded that at present, there is lack of evidence to suggest that antithyroid treatment might ameliorate dementia.

## 5. Thyroid Status, Depression, and Disability

Some studies have shown an association of subclinical thyroid disease with depression [57, 65]. In the Leiden 85+ study which studied individuals > 85 years in age, no consistent association between thyroid status and disability or depressive symptoms was detected [37]. Another observational study also showed that SCH is not associated with metabolic derangement, cognitive impairment, depression, or poor quality of life (QoL) in elderly subjects [61]. A recent study too did not support deleterious effects of subclinical thyroid disorders on physical or cognitive function, depression, or mortality in an older population [62]. Analysis of individuals aged 70−79 years from the Health, Aging, and Body Composition Study showed that higher TSH may be associated with a slight functional mobility advantage [69]. The RCT by Jorde et al. concluded that in SCH where the serum TSH level is in the 3.5–10.0 mIU/litre range, there is no neuropsychological dysfunction or increased symptoms of hypothyroidism compared those in with healthy controls [66]. 

## 6. Thyroid Status and Longevity

There have been few studies exploring the effect of thyroid disease on mortality and longevity in the elderly population. It is important to point out that these results differ from studies in younger population, and they should not be extrapolated to them. 

As mentioned earlier, the Leiden 85+ study showed that higher TSH concentrations and lower free thyroxine levels were associated with a survival benefit [37]. In this study, participants with low levels of TSH at baseline had highest mortality rate, and participants with high TSH levels and low FT4 levels had the lowest mortality rate. The authors speculated that lower thyroid function may lead to lower metabolic rate which in turn could cause caloric restriction. Lower metabolic rate and caloric restriction have both been shown to be associated with improved survival in several animal studies [70–72].

Atzmon et al. conducted their study in elderly Ashkenazi Jews with the median age of study population being 98 years [73]. They demonstrated that centenarians have significantly higher median serum TSH concentrations compared with younger Ashkenazi controls (median age 72 years) and in a population of thyroid disease-free individuals (median age 68 yr) from the US National Health and Nutrition Examination Survey 1998–2002. Similar results were noted in the study by van den Beld et al. who showed that low serum free T4 was associated with a better 4-year survival in men aged 73 to 94 years [74]. The study by de Jongh et al. showed no difference in mortality in elderly population with subclinical thyroid disorders [62]. Another recent study in men aged > 65 years concluded that there was no beneficial or detrimental effect of subclinical thyroid dysfunction in older men [75]. On the other hand, various studies have shown subclinical hyperthyroidism to be associated with higher mortality or no association with mortality. The studies by van den Beld et al. [74] and de Jongh et al. [62] have not shown any change in mortality in their subjects with subclinical hyperthyroidism. Previously, Parle et al. showed that a single measurement of low serum thyrotropin in individuals aged 60 years or older is associated with increased mortality from all causes, and in particular mortality due to circulatory and cardiovascular diseases [38]. Similar results were found by Collet et al. [43] that endogenous subclinical hyperthyroidism is associated with increased risks of total as well as IHD mortality, with highest risks of CHD mortality and AF when TSH level is lower than 0.10 mIU/litre. 

These results are not entirely unexpected as similar results have been shown in animal studies. It has been reported that rats in which hyperthyroidism was induced had a shorter life span [76, 77]. Wistar rats with induced hypothyroidism had a longer life span than euthyroid rats [78]. Ames and Snell dwarf mice have low levels of prolactin, growth hormone, and thyroid hormones and have both reduced body core temperature and metabolic rates consistent with hypothyroidism and live 40–70% longer than euthyroid mice [79].

There have been few recent studies exploring the longevity with raised TSH and familial/genetic basis for this phenomenon. Rozing et al. in Leiden Longevity Study showed that when compared with their partners, the group of offspring of nonagenarian siblings showed a trend toward higher serum TSH levels in conjunction with lower free T4 levels and lower free T3 levels [80]. In their extension to this study, they found that lower mortality in the parents of nonagenarian siblings was associated with higher serum TSH levels, lower free serum T4 levels, and lower free T3 levels in the nonagenarian siblings [81]. In the study of Ashkenazi Jews, a heritable phenotype characterized by raised serum TSH which is associated with human longevity was identified. Carriers of rs12050077 and rs10149689 single nucleotide polymorphism in the TSH receptor gene had higher serum TSH, possibly contributing to decreased thyroid function and longevity [82].

## 7. Thyroid Status and Bone Health

In 1891, von Recklinghausen reported a “worm eaten” appearance of the long bones in a young woman who died from hyperthyroidism [83]. Thyroid hormone affects bone calcium metabolism by direct action on osteoclasts or by acting on osteoblasts which in turn mediate osteoclastic bone resorption [84]. TSH may also have a direct effect on bone formation and bone resorption, mediated via the TSH receptor on osteoblast and osteoclast precursors [85]. Various studies have examined the association of thyroid function with bone mineral density (BMD) and/or fracture risk. In the Tromsø study, the subjects with serum TSH below the 2.5 centile had significantly lower BMD as compared to that of controls with serum TSH in the normal range. Conversely, the postmenopausal women with serum TSH above the 97.5 centile had significantly higher BMD at the femoral neck than women with serum TSH in the normal range [86].

A study from Japan, using quantitative ultrasound, demonstrated that subclinical hypothyroidism does not affect bone turnover but has an impact on bone structure [87]. In the Study of Osteoporotic Fractures in women > 65 years, low TSH was not associated with BMD or accelerated bone loss in older ambulatory women [88]. However, the study group, analysing data from the same cohort, also showed that low serum TSH levels were associated with increased risk for new hip and vertebral fractures [89]. Another study in men > 65 years showed that patients with subclinical hyperthyroidism or hypothyroidism were at increased risk of hip fracture [90]. The recent MrOS study concluded that although neither TSH nor FT4 are associated with bone loss, lower serum TSH may be associated with an increased risk of hip fractures in older men [91]. There has been two meta-analyses on the effects of long-term thyroxine replacement [92, 93]. Both of these showed that levothyroxine therapy leading to suppressed TSH was associated with significant bone loss in postmenopausal women but not those that were premenopausal. 

## 8. Conclusions and Recommendations

There is increasing wealth of data suggesting that serum TSH levels increase with age, particularly after 70 years. This might reflect reduced end organ sensitivity, reduced turnover, and clearance, a genetic trait conferring a survival benefit or a combination of factors. In addition, no clear benefit is seen in treating a high TSH on a multitude of outcomes in the elderly. In fact, there is a possibility that treatment of SCH in the very elderly may lead to adverse outcomes. On the other hand, a low TSH has been associated with worse outcomes in the older age group. However, to confirm these findings, large scale intervention trials assessing mortality, cardiovascular events, mood, disability, fractures, and cognition are required. This could then lead to the use of age-specific TSH reference ranges.

## 9. Summary of Recommendations—For Elderly Population Only

- Thyroid function results in the elderly (>70 years) should be interpreted with caution as the “normal” range may be different from that in younger population.
- A single abnormal TSH result should be monitored over time as a substantial number of people with subclinical thyroid disease will normalise spontaneously.
- Subclinical hypothyroidism may be associated with adverse cardiac outcome if TSH is > 10 mU/litre, but there is no evidence that it has any adverse cardiac effect in people with TSH < 10 mU/litre.
- Subclinical hyperthyroidism is associated with increased incidence of heart failure, AF, IHD, and mortality, especially if TSH is < 0.1 mU/litre.
- There is conflicting evidence linking subclinical hypothyroidism with cognitive impairment, but more recent larger studies are against any association or benefit with intervention.
- Subclinical hyperthyroidism is associated with higher incidence of cognitive impairment, but at present, there is no evidence to support use of antithyroid drugs for dementia.
- There appears to be no association of subclinical thyroid disease with depression and physical disability.
- Subclinical hypothyroidism appears to lead to a survival benefit in the elderly population.
- Subclinical hyperthyroidism appears to be associated with higher mortality.
- Subclinical hypothyroidism in elderly people should not be treated routinely if TSH is < 10 mU/litre and if the patient is otherwise well.
- Subclinical hyperthyroidism on the other hand should be evaluated further especially regarding other cardiovascular risk factors and comorbidities, and then a decision should be made regarding treatment, especially in people with TSH < 0.1 mU/litre.
